# Effect of inorganic carbonate and organic matter in thermal treatment of mercury-contaminated soil

**DOI:** 10.1007/s11356-021-14024-z

**Published:** 2021-04-26

**Authors:** Kanghee Cho, Jinkyu Kang, Songbae Kim, Oyunbileg Purev, Eunji Myung, Hyunsoo Kim, Nagchoul Choi

**Affiliations:** 1 Research Institute of Agriculture and Life Sciences, Seoul National University, Seoul, 08826 Korea; 2 Department of Energy and Resource Engineering, Chosun University, Gwangju, 61452 Korea

**Keywords:** Mercury, Inorganic carbonate, Thermal treatment, Desorption, Temperature

## Abstract

Thermal treatment of mercury (Hg)-contaminated soil was studied to investigate the desorption behavior of Hg at different temperatures. The soil samples were collected from two locations with different land uses around the mine and industrial site. The effect of soil properties such as inorganic carbonate minerals and organic matter content on Hg desorption was investigated to understand the thermal desorption process. The effect of soil composition on Hg desorption showed that behavior at 100 °C was similar, but a different behavior could be found at 300 °C. The thermal desorption efficiency at 300 °C is affected by the thermal properties of soils and the Hg desorption capacity of the soils. The Hg from both soil types was removed above 300 °C, and Hg was effectively removed from mine soil due to the partial decomposition of carbonate in the soil composition, while industrial soil showed that desorption would be restrained by Hg organic matter complexes due to organic matter content. Despite a relatively higher concentration of Hg in the mine soil, Hg removal efficiency was greater than that in the industrial soil. Sequential extraction results showed that only the Hg fractions (residual fractions, step 6) in mine soil changed, while the industrial soil was affected by changes in Hg fractions (step 3 to step 6) at 300 °C. Changes in soil pH during thermal desorption are also influenced by heating time and temperature. Therefore, the mechanisms of Hg desorption during thermal treatment were observed by soil properties. The volatilization of Hg in the soil is induced by organic carbon, while soil Hg release is controlled by organic matter complexes.

**Supplementary Information:**

The online version contains supplementary material available at 10.1007/s11356-021-14024-z.

## Introduction

Mercury (Hg) contamination has become an environmental problem and attracted increasing attention (O’Connor et al. 2019; Wang et al. 2019). Sites contaminated with Hg are widespread due to human activities such as mining and coal combustion, leading to soil contamination. Almost all Hg compounds are toxic and can be dangerous at very low levels in the environment. Exposure to Hg has been reported to cause diseases (Feng and Qiu 2008). Hg-contaminated soil is considered a hazard due to the toxic characteristics of Hg and the fact that it can be readily taken up by agricultural crops (Li et al. 2019). When Hg gets into a food chain, it damages the nervous systems and reproductive systems of mammals, including humans. Because Hg is a persistent substance, it can build up. Hence, Hg can accumulate in the human body through the food chain, and this could be an even more serious problem in the future (Zhao et al. 2019).

In contaminated soils, soil components play important roles in regulating contaminant retention, which influences the mobility, solubility, and toxicity of contaminants in the soil. Among the soil components, O, N, and S, which contain functional groups of organic ligands involved in the soil organic matter, are considered important in immobilizing contaminants due to their stable complex formation (Vasques et al. 2020). In the soil, Hg is mostly bound to mineral surfaces and organic matter. Overall, the various forms of Hg species including HgCl_2_, HgS, and HgO are affected by the organic matter in soil (Wang et al. 2012; Park et al. 2015), especially high molecular weight hydrophobic compounds or hydrophobic neutral organic matter (Stevenson 1994; Li et al. 2019). According to the hard and soft acids and bases (HSAB) principle, Hg retention in soil is controlled by organic matter functional groups, which thereby play an important role in soil-Hg binding (Riccardi et al. 2013; Reis et al. 2016). Because of the interactions between Hg and soil components, Hg can bind more strongly with soil constituents than many other metals in soil (Tomiyasu et al. 2017). Unfortunately, Hg-contaminated soils, owing to their different characteristics, have caused substantial challenges in soil remediation. Therefore, the soil in the Hg-contaminated sites must be treated by appropriate remediation techniques to prevent contamination problems.

Contaminated soils have traditionally been treated with remedial techniques (Jho et al. 2015; Huang et al. 2019; Cho et al. 2020), such as soil washing, solidification/stabilization, thermal desorption, and phytoremediation, which aim to remove contaminants from soil to achieve target cleanup thresholds. Among them, many researchers have reported the feasibility of Hg remediation with thermal treatment (Rumayor et al. 2013; Reis et al. 2015). Generally, thermal treatment can be considerably influenced by soil properties. To increase the heat mass transfer efficiency, there is a need to consider thermodynamic Hg speciation and Hg transfer between the solid and gas phases. The saturated vapor pressure of soil minerals with increasing temperature is likely to affect thermal desorption, which is related to thermal vaporization and desorption kinetics. During the thermal treatment, the increased vapor pressures from soil compositions can destroy the force existing between soil and Hg, which improves the thermal transfer capability, facilitating Hg desorption, vaporization, and removal by induced gas flow (Falciglia et al. 2011; O'Brien et al. 2018). However, Hg sorption onto the soil matrix can occur as nonspecific or specific adsorption, forming inner-sphere complexes, such as specific adsorption, hindering Hg desorption with increasing temperature. Because of such binding, soil minerals, and the amount and type of organic matter in the soil can significantly affect the transition from the soil to the gas phase of Hg. Therefore, thermal desorption is controlled by soil properties and multiple desorption mechanisms such as film diffusion, chemical desorption, and intra-particle diffusion (Li et al. 2020).

Several parameters have been associated with the efficiency of the thermal desorption for soil contaminated with Hg, in terms of temperature, heating rate, reaction time, and chemical additives (Guo et al. 2017; Lee et al. 2017; Liu et al. 2019; Tian et al. 2019). In the literature, there is no unique practice for thermal desorption. In recent years, most studies have investigated the thermal treatment removal efficiency of Hg at various temperatures and focused on how to decrease the temperature when thermal treatment with additives such as FeCl_3_ and SnCl_2_ was applied (Ma et al. 2014; Lee et al. 2020). However, thermal treatment with additives is likely to affect soil properties that are related to soil qualities because a large amount of additive is still required, and post-treatments are still necessary. In addition, the additive remains in the soil for a long time. Additionally, many studies principally analyzed the effects of initial soil conditions on thermal desorption efficiency with N_2_ gas flow, but little attention has been paid to the effects of change of soil properties during thermal desorption process. In this study, we investigated the remediation of two soils contaminated by Hg using thermal treatment under air, and the objectives of this study were to evaluate the influence of soil composition on Hg removal and to investigate the behavior of Hg at different temperatures. Changes in soil properties during thermal treatment were also investigated by heating time and temperature.

## Materials and methods

### Hg-contaminated soil

Soil samples were collected from each of the two locations with different land uses around the mine and industrial site of Guizhou province, China. The soil samples were air-dried and passed through a 2-mm sieve. To investigate the chemical composition of the soils, atomic-absorption spectrophotometry (AAS, AA-7000, Shimadzu, Japan) and X-ray fluorescence (XRF, S4 PIONEER, Bruker AXS, Germany) were performed. The total concentrations of Hg in 1-g soil samples were extracted using HCl and HNO_3_ at a 3:1 ratio (i.e., aqua regia).

A six-step sequential extraction proposed by Hall and Pelchat (2005) was conducted for Hg extraction from contaminated soils, which included soluble and exchangeable (step 1), labile organic components (step 2), amorphous oxide-bound (step 3), crystalline oxide-bound (step 4), non-labile organic and elemental (step 5), and sulfide-bound and residual (step 6) fractions. A detailed description of the sequential extraction procedure used in this study is summarized in Table S1.

### Thermal treatment of Hg-contaminated soil

A series of 10-g samples were taken in a pre-weighed quartz boat, pyrolyzed in an electric furnace under an air atmosphere at different temperatures. A heating rate of 10 °C/min was performed in an electric furnace to reach the specific temperature and residence times. Once the electric furnace reached the expected temperature from 100 to 700 °C, the sample was inserted into an electric furnace for 30 min. After the experiment, the contents were cooled to ambient temperature. To investigate the effect of soil properties, Hg sequential extraction of the thermally treated soils was conducted. After each sequential extraction step, the solid residues were recovered, and the residues were examined comparatively with bulk soils via X-ray diffraction (XRD, X’Pert Pro MRD, Panalytical, The Netherlands) and Fourier transform infrared (FTIR) spectroscopy (Nicolet 6700, Thermo Fisher Scientific, USA). Moreover, extraction of water-soluble organic matter was conducted in order to describe the decomposition of the organic matter. The total organic carbon (TOC) of soil organic matter in soil was extracted by adding 15 mL of water to 2.0 g of soil in a 50-mL conical tube. TOC content was measured using a TOC analyzer (TOC-V SCN, Shimadzu, Japan) without further dilution. Hg removal efficiency was calculated according to the following expression:

1 $$ R\left(\%\right)=\frac{C_0-C}{C_0}\times 100 $$

where *C*_0_ is the initial contaminant concentration in soil (mg/kg) and *C* is the residual contaminant concentration in soil after the thermal treatment (mg/kg).

### Kinetic experiments of Hg-contaminated soil

The Hg removal kinetics of the two soil samples were also investigated at 100 and 300 °С for different times in the range of 10–60 min. Experiments were performed in duplicate; for each selected temperature, weight and pH difference were compared according to the pyrolytic conditions. An exponential decay kinetic model (Falciglia et al. 2011) was applied to evaluate the experimental data. The kinetic equation can be expressed by Eq. (2) as follows:

2 $$ {C}_t={C}_0{e}^{-K{t}^n} $$

where *C*_*t*_ is Hg concentration in soil (mg/kg) after a treatment time *t* (min), *C*_0_ is the initial Hg concentration in soil (mg/kg), *K* (min^−1^) is the rate of decay of the function, and *n* is a parameter that represents the non-linearity of thermal desorption kinetics.

### Characterizations

The thermo-gravimetric behavior of the sample was analyzed with a thermo-gravimetric analyzer (TGA, SDT Q600, TA Instruments, USA) in an air environment. The thermo-gravimetric behavior of the sample was analyzed at a heating rate of 10 °C/min under air, and the scan range was approximately 900 °C. Particle size analysis was conducted using a laser particle size analyzer (Mastersizer 2000, Malvern, UK). The pH of the samples was analyzed by mixing with deionized water at a ratio of 1:5 (soil:deionized water) using a pH meter (D-71, Horiba, Japan). The organic carbon, nitrogen, and sulfur contents were analyzed using an elemental analyzer (VarioEL, Elementar, Germany). The organic matter content was determined using the ignition method (Heiri et al. 2001). In this test, 5 g of the soil sample was heated for 1 h at 400 °C, and the weight loss was determined and assumed to represent the organic matter content in the soil. The samples were subjected to XRD analysis. Cu Kα radiation was used at an acceleration voltage of 40 kV and a current of 30 mA. The 2θ section from 10 to 70° was analyzed for the soil. The functional groups of samples were characterized using a Fourier transform infrared spectrometer. To reveal the elemental composition of soils used in the present study, both soil types were analyzed by field emission scanning electron microscopy (FE-SEM, S4800, Hitachi, Japan) with an energy dispersive spectrometer (EDS).

## Results

### Characterization of Hg-contaminated soil

The physicochemical characterization of the soil samples is summarized in Table 1, and the mine and industrial soils show different chemical compositions. The organic matter in the mine and industrial soils was 1.90% and 8.60%, respectively. Higher organic carbon content was observed in the mine soil. Both study soils were primarily contaminated with Hg. The concentration of Hg in each sample was 1049.2 ± 15.3 (mine soil) and 38.3 ± 2.18 (industrial soil) mg/kg, respectively. The minerals found by the XRD analysis of mine soil were calcite, dolomite, and quartz, while muscovite and quartz were observed as the major minerals in the industrial soil. In addition, the main chemical compositions of the mine soil are dolomite and quartz, in which SiO_2_, CaO, and MgO are dominant, while the industrial soil is primarily associated with SiO_2_, Al_2_O_3_, and Fe_2_O_3_. The pH of mine soil was higher than that of industrial soil, which could be because of carbonate minerals such as dolomite and calcite. The elemental distribution of the soil particles was explored by SEM-EDS (Fig. S1), and Hg was visible in back-scattered electron (BSE) images as a white halo on light gray particles. The SEM images of both soils show that the Hg has been adsorbed to the mineral surfaces. The EDS elemental spectra analysis showed that the industrial soil was composed of Al, Si, and O, while the mine soil was composed of C, Ca, Mg, Si, and O. It can be seen that Hg is mainly incorporated into the carbonate and silicate matrix.

**Table 1:** Physicochemical properties of soil samples

Parameters	Mine soil	Industrial soil
pH	7.46	6.55
Organic matter (%)	1.90	8.60
Sand (%)	26.7	16.0
Silt (%)	71.3	81.8
Clay (%)	2.0	2.2
Organic C (%)	9.21	1.04
N (%)	0.13	0.16
S (%)	0.15	0.47
Hg (mg/kg)	1049.2	38.3
SiO_2_ (wt%)	36.9	54.0
CaO (wt%)	33.4	0.42
MgO (wt%)	15.8	1.17
Al_2_O_3_ (wt%)	7.71	26.9
Fe_2_O_3_ (wt%)	3.51	13.3
SO_3_ (wt%)	0.19	0.10

**Fig. 1:**
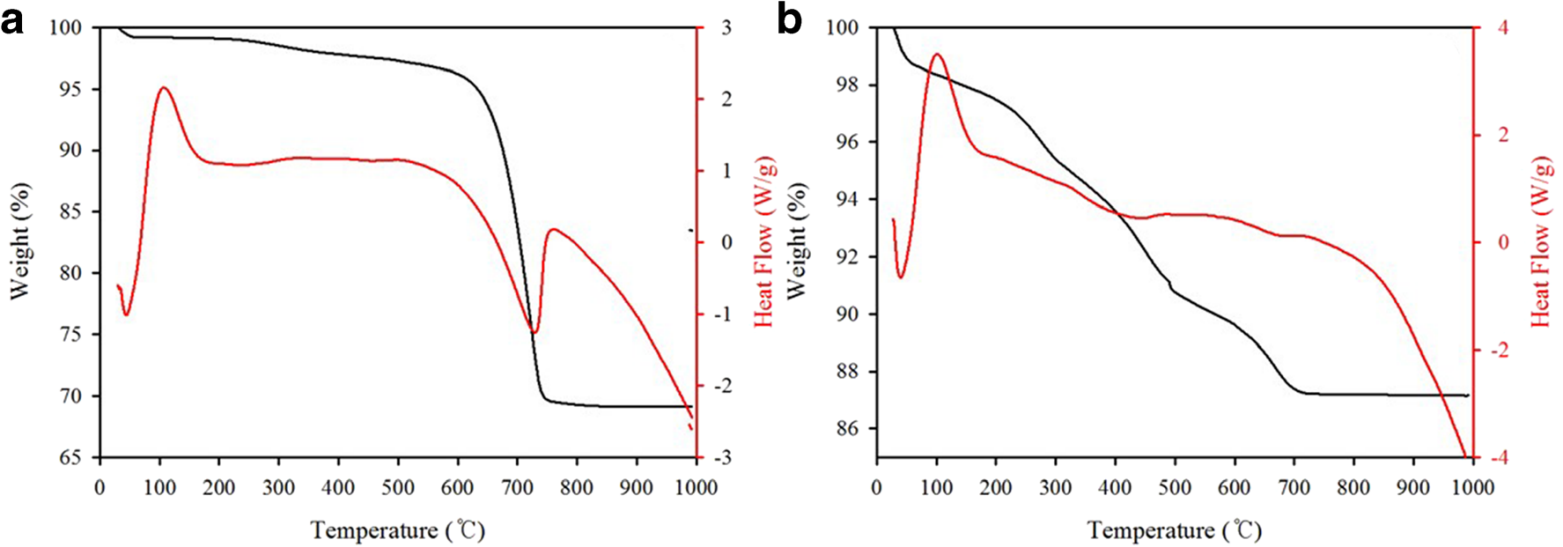
TG and DSC curves of **a** mine soil and **b** industrial soil

The TG and DSC curves of the soil at a heating rate of 10 °C/min in air are shown in Fig. 1a–b. In the mine soil, a stage of sharp mass loss between 600 and 750 °C was found in the high-temperature region. This peak is approximately 26.5% of the mass loss. This endothermic peak is related to the thermal decomposition of dolomite. The breakdown of dolomite in air could be from 650 to 700 °C (Qian et al. 2019), and the products of dolomite thermal decomposition are calcite (CaO) and periclase (MgO). Then, calcite decomposition occurs subsequently at a higher temperature (Valverde et al. 2015). The decomposition process of dolomite can be expressed as Reactions 3 and 4.

3 $$ \mathrm{CaMg}{\left({\mathrm{CO}}_3\right)}_2\to {\mathrm{CaCO}}_3+\mathrm{MgO}+{\mathrm{CO}}_2 $$

4 $$ {\mathrm{CaCO}}_3\to \mathrm{CaO}+{\mathrm{CO}}_2 $$

The XRD intensity of the dolomite peak was also observed to decrease (Fig. 4a). Contrarily, four major weight losses were found in the industrial soil. These are caused by water volatilizing, humic substances cracking, and the various forms of Hg species (Soucémarianadin et al. 2019; Li et al. 2020). This suggests that the Hg in the soil may have been sorbed to the matrix mineral surfaces, or to organic matter. However, the precise species could not be distinguished. In this soil, it was revealed that the organic matter content was relatively high (Table 1). Therefore, Hg would likely be associated with organic matter complexes in the industrial soil.

### Hg desorption efficiency at various temperatures

**Fig. 2:**
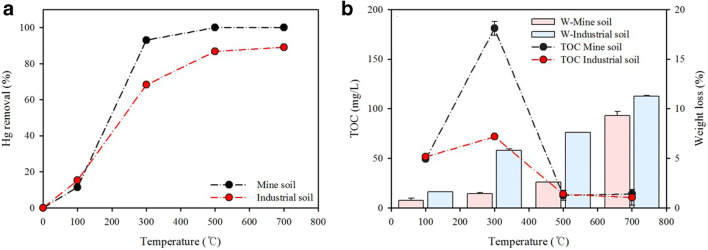
**a** Hg removal from soil samples at different temperatures; **b** TOC concentrations (TOC) and weight loss (W) of soil samples treated at different temperatures

The Hg desorption efficiency for both soils is shown in Fig. 2a and shows the behavior of Hg at different temperatures. The soil Hg desorption began to increase with increasing temperature, and Hg from the mine soil could be completely removed at 500 and 700 °C, respectively. The desorption efficiency of the industrial soil under the same conditions was only 86.7% and 89.1%, respectively. Considering that the organic matter content of the industrial soil is higher than that of the mine soil, a possible explanation for this result is that more energy is required to remove Hg from industrial soil (Falciglia et al. 2011; Lee et al. 2020). Despite a relatively higher concentration of Hg in the mine soil, Hg desorption efficiency was greater than that in the industrial soil. These results are consistent with the results of the TOC concentrations and weight loss of the treated soil at different temperatures in Fig. 2b. The TOC concentrations of the treated soil at 100–300 °C were higher than that of untreated soil, which is mainly due to the break-up of organic carbon, while the TOC concentration was reduced as the temperature increased from 300 to 700 °C. These findings suggest that soil organic carbon contributes to an increase in the gas phase of Hg. Weight loss was also affected by organic matter content (up to ~ 500 °С).

**Fig. 3:**
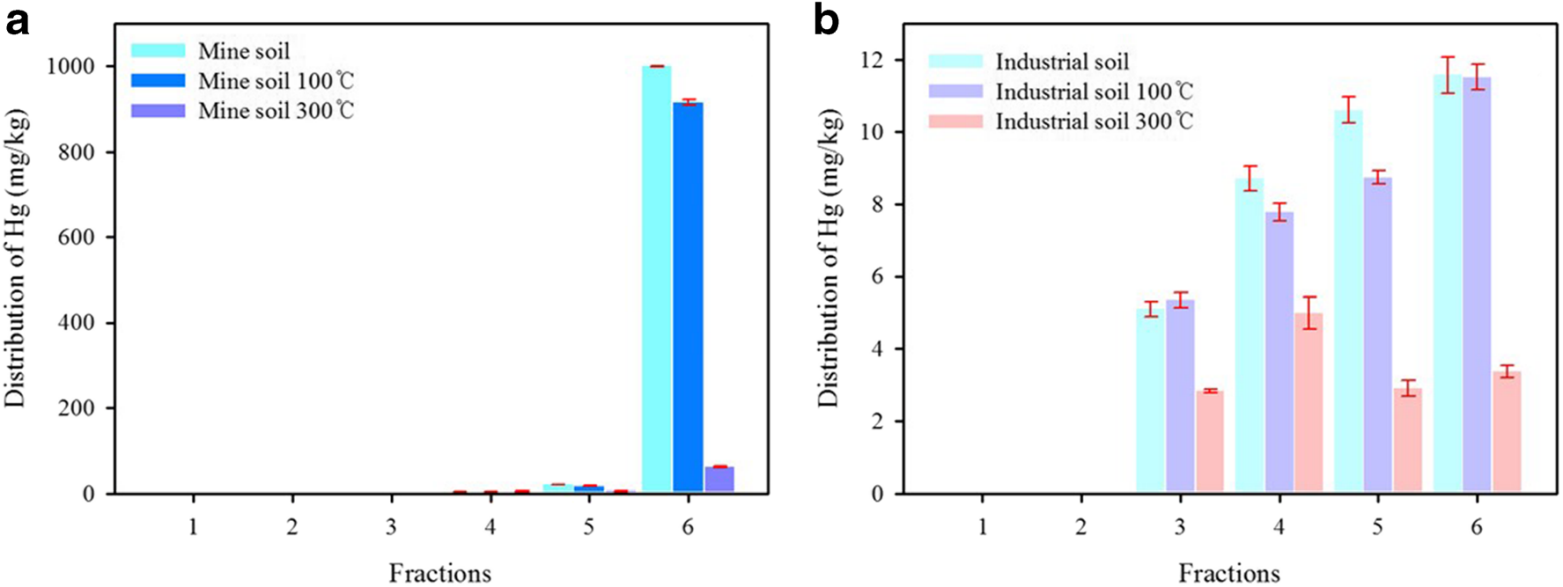
Sequentially extracted fractions of Hg from Hg-contaminated soil samples before and after thermal treatment at 100 and 300 °C, for 30 min (**a** mine soil and **b** industrial soil)

A six-step sequential extraction was conducted to assess the relative lability in the untreated original soils (Fig. 3). Neither soil released Hg in fractions 1 and 2. This result implies that the original soils were less mobile in the environment. The Hg in the industrial soil was bound to amorphous oxide-bound (step 3, 14.2%), crystalline oxide-bound (step 4, 24.2%), non-labile organic and elemental (step 5, 29.5%), and sulfide-bound and residual (step 6, 32.1%), which indicates that each step reflects the presence of various Hg compounds in the soil (Biester and Nehrke 1997; Tong et al. 2011). Meanwhile, the Hg in mine soil was primarily associated with the sulfide-bound and residual (step 6, 97.8%). In general, the mobility of Hg in soil decreased as the sequential extraction to the later fractions. It can be seen that Hg in the industrial soil was weakly bound to soil and more labile compared to Hg in the mine. Sequential extraction was also subjected to thermally treated soil. After both soils were thermally treated at 100 °C for 30 min, the sequential extraction trend was similar to that of the untreated soils. Although Hg residual fractions (step 6) in the industrial soil were not removed, the Hg residual fractions (step 6) of the mine soil were reduced to 8.40%. This finding indicates that the change in Hg residual fractions (step 6) at 100 °C is associated with the form of soil properties. It can be seen that organic carbon was holding Hg in a more labile form, whereas functional groups of organic matter were associated with immobile fractions with tighter binding. This indicates that the high Hg-binding capacity depends on soil organic matter content. After treatment at 300 °C for 30 min, the concentration of Hg residual fractions (step 6) extract was 61.5 mg/kg with a removal rate of 93.8%. It was found that the removal efficiency increased as the temperature rose, and the desorption efficiency of mine soil at 300 °C was higher than that of industrial soil. It can be seen that the difference in removal efficiency at 300 °C may be related to the change in mineralogical properties. Meanwhile, the various Hg species in the soil could not be distinguished by sequential extraction. Hg adsorbed to organic matter is simultaneously associated with different forms and is therefore difficult to separate (Biester and Scholz 1996; Kim et al. 2000; Sysalova et al. 2017). It can be seen that the sequential extraction proposed by Hall and Pelchat (2005) is limited to determining Hg species and influenced by the amount of carbonates and soil organic matter.

### Characterization of Hg-contaminated soil at different temperatures

**Fig. 4:**
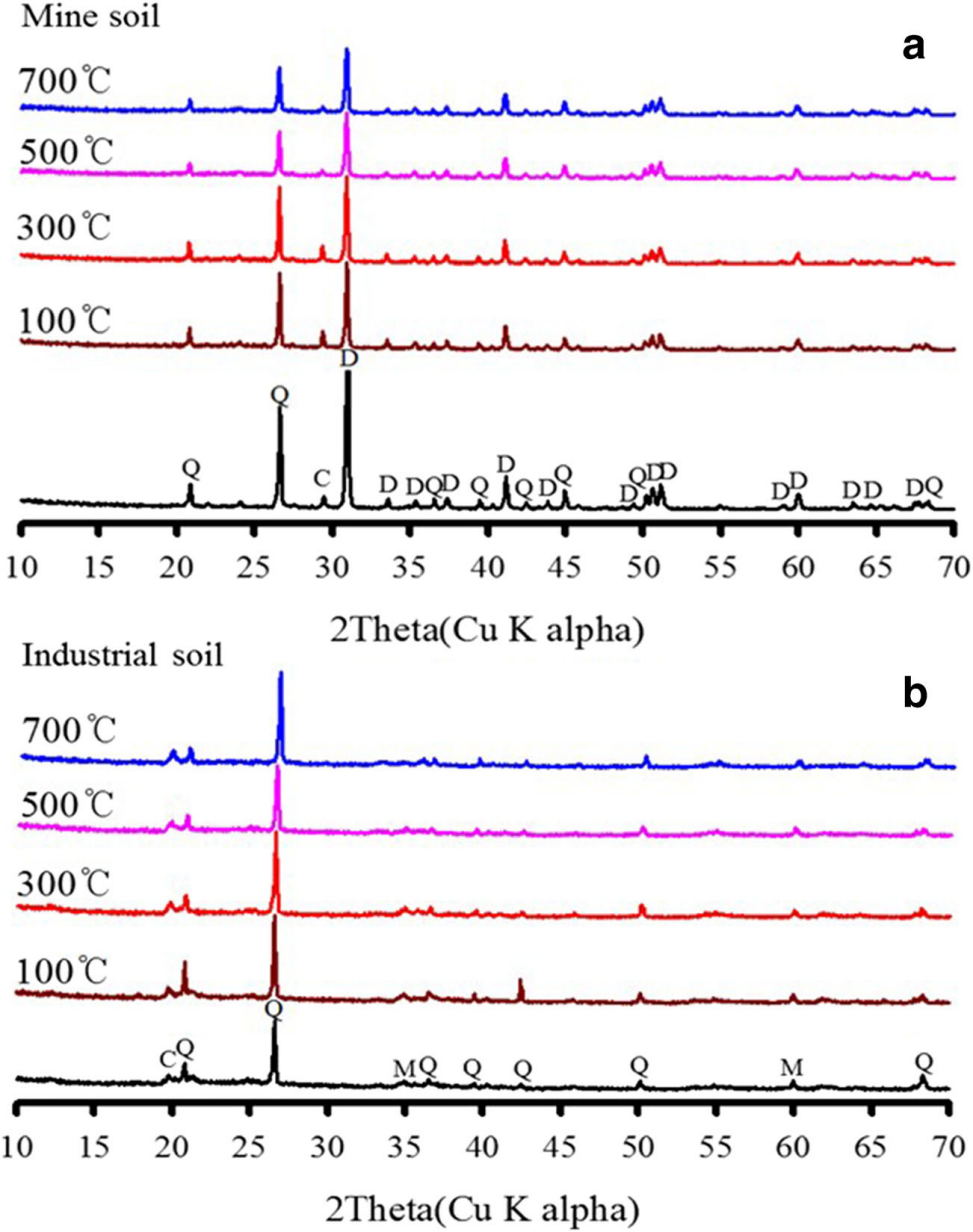
X-ray diffraction patterns of Hg-contaminated soil samples before and after thermal treatment at 300 and 700 °C, for 30 min (**a** mine soil and **b** industrial soil) (C, calcite; D, dolomite; M, muscovite; Q, quartz)

To study the soil minerals’ changes during thermal treatment, XRD analyses were conducted to confirm the changes (Fig. 4). By comparing the patterns of untreated and treated samples, no significant changes were observed in the treated soil. However, the intensity of the dolomite peaks decreased in the mine soil, indicating that carbonate components with Hg in the soil may be removed from the soil into the gas phase. It can be seen that partial decomposition of dolomite in the soil was considered for the displacement of the gas phase of Hg. An XRD analysis was conducted for the residual soils after sequential extraction (Fig. S2). The XRD peak of the industrial soil did not show any distinct change from the XRD pattern of the untreated original soil. However, the dolomite peak in the mine soil decreased and disappeared as the sequential extraction proceeded to the later fractions; only quartz was observed in the residual fractions. It was found that correlations between dolomite and Hg were observed, which indicates that the dolomite influenced Hg removal in the soil.

**Fig. 5:**
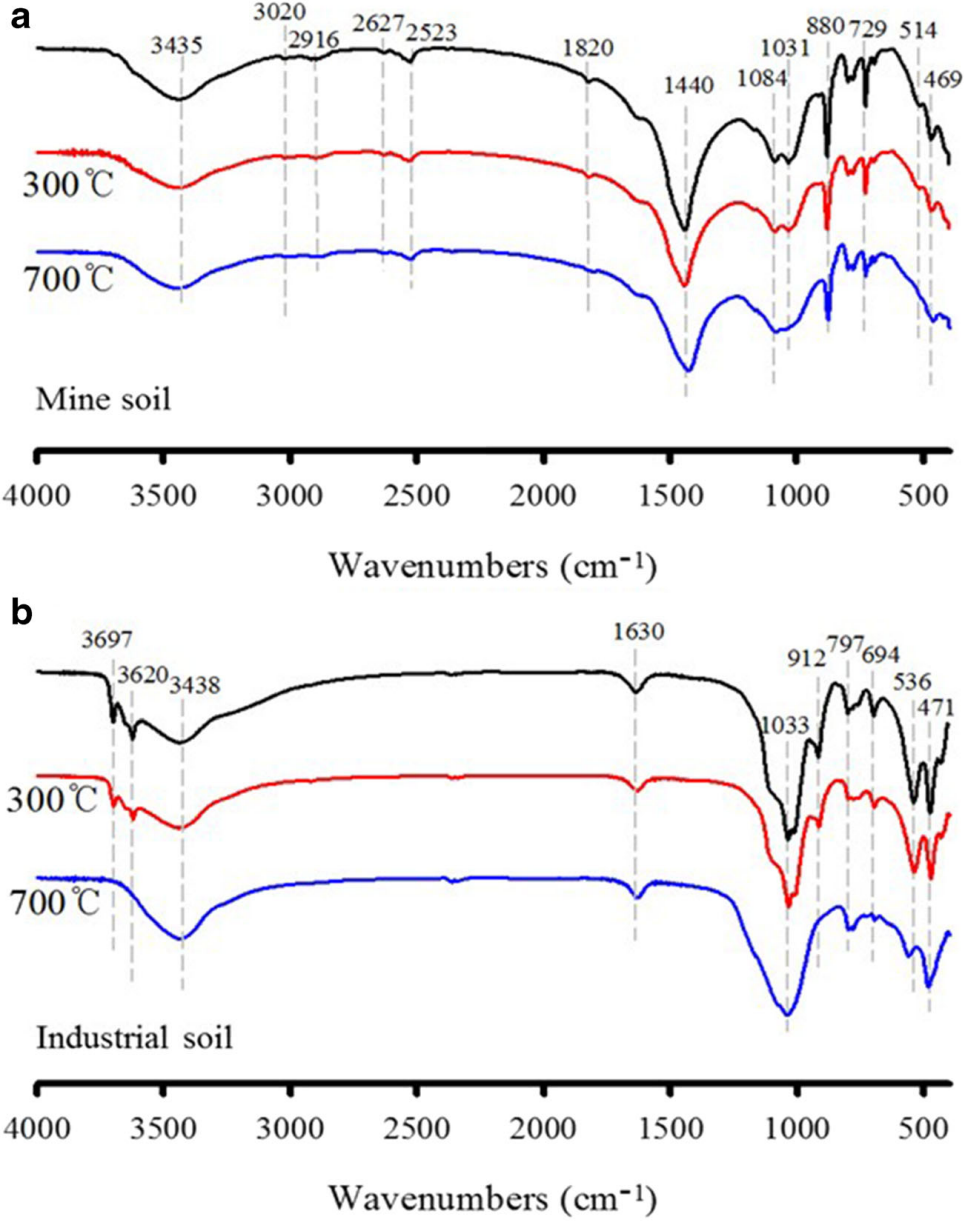
FTIR spectra of Hg-contaminated soil samples before and after thermal treatment at 300 and 700 °C, for 30 min (**a** mine soil and **b** industrial soil)

The thermal decomposition of soil components during thermal treatment was further explored, and the results of the functional groups of the heating conditions detected by FTIR spectroscopy are shown in Fig. 5. Hg binds to organic matter colloids because of its high specific surface area and the presence of surface functional groups (Yang and Ok 2017; O’Connor et al. 2019). Both soils showed characteristics of organic matter functional groups such as hydroxyl and carboxyl. The broad bands in the 3400–3300 cm^−1^ region, the band near 3700 and 3620 cm^−1^, and the peaks at 913 and 536 cm^−1^ are attributed to the vibration of hydroxyl groups (Huang et al. 2019). In addition, peaks at 1820, 1630, and 1440 cm^−1^ correspond to the characteristics of carboxyl groups (Wang et al. 2019). In industrial soil, peaks at 1033, 912, 797, 694, and 471 cm^−1^ are dominated by Si–O stretching vibration bands of quartz (Kim et al. 2015). Furthermore, the carbonates in the mine soil were demonstrated by the absorption bands between 3020 and 2916 cm^−1^, 2627 and 2523 cm^−1^, and 1084 and 1031 cm^−1^ (Ji et al. 2009).

When the temperature rose to 300 and 700 °С, the interaction between Hg and soils was investigated and compared with the FTIR results of the soil. With increasing temperature, the stretching vibration absorptions of carbonate species weakened gradually in the mine soil. This could be explained by the decomposition of carbonates. Therefore, it seems that the soil sample has a considerable amount of dolomite, which undergoes various thermal reactions such as oxidation and decomposition during thermal treatment. At the same time, the surface of particles became more porous along with the release of the CO_2_ (Reactions 3 and 4). This implies that the Hg in the sample may have been removed to the matrix mineral surfaces, or to organic matter. Meanwhile, in the industrial soil, hydroxyl compounds at 3697 and 3620 cm^−1^ decrease due to the interaction of functional groups of organic ligands with Hg. The bands at 3438 and 1630 cm^−1^ in the industrial soil were the most prominent, but these bands did not show any distinct change. The two soils show the interaction of Hg with hydroxyl groups and between Hg and carboxylate functional groups. These peaks still appear but in a weakened form at 300 °C, and almost completely disappear at 700 °C. Therefore, it seems that a high removal of Hg in the soil could be achieved by thermal desorption.

Moreover, changes in various functional groups of the residual soils after sequential extraction for each fraction were evaluated (Fig. S3). Compared with residual soil from mine soil for each fraction, new peaks at 3695 and 3619 cm^−1^ arise in the FTIR curve of step 3 and step 4 residual soil, which are attributed to the hydroxyl groups. Notably, the hydroxyl groups were observed in different steps at 100 and 300 °C, which may be related to the thermal desorption behavior. It can be seen that Hg compounds in the mine soil have no tendency to react with weak acids or bases and are immobile on the solid matrix. This result provides evidence that Hg binding depends on soil characteristics such as inorganic carbonate minerals. In contrast to mine soil, Hg retention capacity in industrial soil may be due to binding with organic matter, which has covalent bonds. It indicates that the strong binding of Hg in soils is due to stable complex formation. Hg in soil tends to be involved in covalent bonds with the O atoms of organic matter functional groups (e.g., –OH, O–C=O, and C=O), implying that organo-Hg compounds were stronger than carbon compounds, such as carbonates (Soares et al. 2015; Wang et al. 2019).

### Removal kinetics of Hg under thermal treatment

**Fig. 6:**
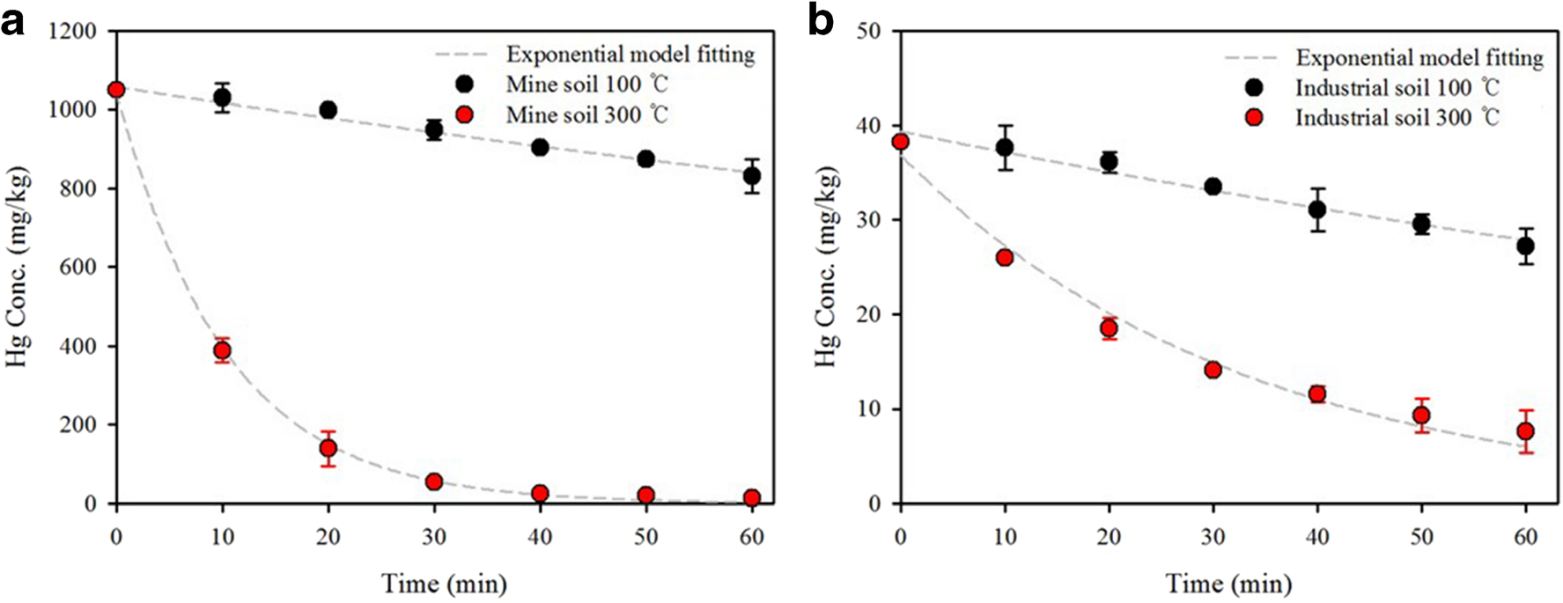
Hg removal efficiency at 100 and 300 °C within 60 min of **a** mine soil and **b** industrial soil

The Hg removal efficiencies of the two soil samples were investigated at 100 and 300 °С (Fig. 6a–b). At each temperature, the removal efficiency increased with reaction time. The residual Hg concentrations at different temperatures were fitted via an exponential decay kinetic model (Eq. 2), and the kinetic parameters are shown in Table S2. The *K* value increased with increasing temperature for soils due to the nature of the thermal process, and the *K* value of the mine soil was higher than that of the industrial soil. No significant difference in either soil type was observed at 100 °C, but a difference in removal efficiency was observed at 300 °C. This indicates that the decay rate is correlated with the characteristics of the soil. As shown in Table 1, more CaO was present in the mine soil than in the industrial soil. This difference in chemical composition in the soil seemed to affect Hg removal. The main reason for this may be the partial decomposition of carbonate minerals in the mine soil at 300 °C, caused by the diffusion of the CO_2_ released from the reaction surface and heat transfer inside the particle. The SEM image clearly shows that the surface of the soil grains yields a much more porous structure due to the diffusion of CO_2_, resulting in the partial decomposition reaction (Fig. S4). Zarghami et al. (2015) reported the effect of steam on the partial decomposition of dolomite. Steam improves the connectivity of the pores inside the dolomite particles, which decreases the diffusion resistance of carbon dioxide produced inside the particle. The Hg was bound to the soil component, either by outer-sphere complexes or by inner-sphere complexes (Reis et al. 2016). The adsorbed Hg in inner-sphere sorption sites of soil needed a relatively higher desorption temperature due to a diffusion process (Falciglia et al. 2011; Park et al. 2020). The pores inside the soil particles of mine soil could increase the heat transfer ability and effective diffusivity. The Hg removal efficiency through this porous layer plays a key role in the mine soil.

Soil pH analysis was conducted for the thermal-treated soil. The mine soil showed near-neutral pH when treated at 100 °С, while that treated at 300 °C resulted in a basic pH. Meanwhile, the pH of the industrial soil was always lower than the pH of the mine soil. The two different soil pH values may be explained by, compared with organic matter content, and soil minerals such as dolomite and calcite. The mine soil, which could be related to the degradation of the organic matter-carbonate complexes, which is in correlation with high Ca content, seems to decrease with increasing soil pH. Industrial soil’s pH was slightly increased in terms of the time at different temperatures, which is caused by the loss of organic acids such as fulvic and humic acids (Ravichandran et al. 1999; Xu et al. 2014). Therefore, soil pH changes during thermal desorption are influenced by heating time and temperature.

## Discussion

Based on the analysis above, Hg tends to be immobilized in the soil due to its affinity for mineral surfaces and bonding to organic matter. In this study, the soil composition played an important role in Hg removal. The thermal desorption-related Hg bonding strength of soils can be largely attributed to the interactions between Hg and soil, which involve sorption to soil organic matter and soil matrices, and the influence of soil properties on Hg speciation (Lesa et al. 2009; Park et al. 2015). However, the precise species could not be distinguished by this study. The Hg phase transition reactions and decomposition of Hg solid in standard enthalpy showed that Hg could be converted to the gas phase by thermal treatment (Table S3). The positive values of enthalpy change (ΔH°) indicate that the Hg desorption reactions are endothermic, which indicates that the higher temperature contributes to the effective Hg removal from contaminated soil. In general, Hg compounds such as HgO and HgS are insoluble and much less volatile than other forms of Hg; therefore, they require larger increases in temperature to achieve Hg removal. It seems that thermal desorption would be affected by different Hg species, which was attributed to the presence of surface functional groups such as O-, N-, and S-containing ligands (Powell et al. 2004). Hence, it is noted that organic matter content may be regarded as an important interaction during thermal treatment. The diffusion barrier of Hg release is affected by soil matrix components, and the higher organic matter in the soil controlled the desorption process, resulting in an increase in the diffusion barrier of Hg release. Moreover, for carbonate, the presence of organic carbon made it possible to evaporate Hg rapidly, which could be related to the steam stripping and morphology effect.

Overall, the soil composition for thermal desorption might strongly influence the desorption of Hg from the soil. It can be seen that the vaporization of Hg occurred because of soil components such as moisture and organic matter, which indicates that Hg thermal desorption could be ascribed to stripping by water distillation and gasification through organic matter cracking and partial decomposition of inorganic carbonate. The effect of soil composition on Hg desorption showed that the behavior at 100 °C was similar, but a different behavior could be found at 300 °C. Hg desorption during the thermal treatment at 100 °C occurred from the soil particle surface and was caused by the effect of water stripping. Meanwhile, at 300 °C, Hg desorption is affected by the saturated vapor pressure of soil substances and the creation of permeability, thus contributing to a higher Hg removal due to the partial decomposition of carbonate in the soil composition.

## Conclusions

This study showed that thermal treatment of Hg-contaminated soil with different soil properties could result in different Hg desorption efficiencies. The thermal desorption efficiency in soils at different temperatures is affected by the thermal properties of soils and the Hg desorption capacity of the soils. Since organic matter forms covalent bonds with organic matter functional groups such as hydroxyl and carboxyl, the desorption process is controlled by diffusion barriers such as interactions between Hg and soil. Overall, the soil properties have an essential effect on the desorption process. The thermal desorption mechanism is strongly influenced by the vapor pressure and the morphological changes in the soils. As the temperature increased, the TOC concentration increased at 100–300 °C, and then decreased above 300 °C. Weight loss was also found to be affected by organic matter content. No significant difference in either soil type was observed at 100 °C, but a difference in desorption efficiency was observed at 300 °C. The results of Hg desorption kinetics show that the desorption process was controlled by soil properties, which may be related to the soil Hg desorption capacity. The volatilization of Hg in the soil is induced by organic carbon, while soil Hg release is controlled by organic matter complexes. Therefore, understanding the effects of the desorption process on soil properties may be considered an effective approach to improving Hg desorption efficiency.

## Supplementary Information

ESM 1 (DOCX 2857 kb)

## Data Availability

All data generated or analyzed during this study are included in this published article (and its supplementary information files).
