# Supplementary material for: Effect of inorganic carbonate and organic matter in thermal treatment of mercury-contaminated soil
Source: Environ Sci Pollut Res Int. 2021 Apr 26;28(35):48184–93. doi: 10.1007/s11356-021-14024-z (PMC8410726; doi:10.1007/s11356-021-14024-z)
Supplement: Supplementary file 1 — (DOCX 2857 kb) [file 11356_2021_14024_MOESM1_ESM.docx]

**Table S1** Sequential extraction procedure for different Hg fractions (Hall and Pelchat. 2005).

| Fraction | Hg fraction | Methods |
| --- | --- | --- |
| F1 | soluble and exchangeable Hg | 40 mL of 0.01 M Ca(NO_3_)_2_, shaken for 1 h |
| F2 | Hg combined with labile organics | 45 mL of 0.1 M Na_4_P_2_O_7_, shaken for 1 h |
| F3 | Hg bound to amorphous Fe/Al oxides | 20 mL of 0.25 M NH_2_OH⋅HCl in 0.25M HCl, water bath at 60 ^o^C for 2 h |
| F4 | Hg bound to crystalline Fe/Al oxides | 30 mL of 1 M NH_2_OH⋅HCl in 25% CH_3_COOH, water bath at 90 ^o^C for 3 h |
| F5 | Hg combined with non-labile organics and elemental Hg | 20 ml of 40% HNO_3_, shaken for 2 h |
| F6 | HgS | 6 mL of 12 M HCl and 2 ml of 16 M HNO_3_, water bath at 90 ^o^C for 3 h |

**Table S2** Desorption parameters (k, n) and correlation coefficient (R^2^) of exponential kinetic model at temperature of 100, and 300 ºС for all tested soils.

| Soil sample | T (ºС) | Exponential decay kinetic model | | |
| --- | --- | --- | --- | --- |
|  |  | K (min^-1^) | n | R^2^ |
| Mine soil | 100 | 0.010 | 0.398 | 0.986 |
|  | 300 | 0.949 | 0.019 | 0.999 |
| Industrial soil | 100 | 0.008 | 0.576 | 0.984 |
|  | 300 | 0.299 | 0.010 | 0.997 |

**Table S3** Mercury phase transition reactions, decomposition of mercury solid, carbonate components in soil and the related thermodynamic values (Park et al. 2015).

| Thermodynamic reactions | Log K (25 ºС) | ΔH° (Kcal mol^-1^) |
| --- | --- | --- |
| Hg_(l)_ = Hg_(g)_ | -5.59 | 14.67 |
| HgCl_2(s)_ = HgCl_2(g)_ | -6.84 | 20.04 |
| HgS_(s)_ = HgS_(g)_ | -13.64 | 27.57 |
| HgO_(s)_ = HgO_(g)_ | -14.41 | 31.70 |
| 2HgO_(s)_ = 2Hg_(g)_ + O_2_ | -31.66 | 72.7 |
| HgO_(s)_ = HgO_(g)_ | -14.41 | 31.7 |
| 2HgO_(s)_ = 2Hg_(g)_ +O_2_ | -2.84 | 9.34 |
| HgSO_4(s)_ = Hg(g) +SO_2 (g)_ + O_2_ | -57.13 | 112.8 |
| 2HgSO_4(s)_ = 2Hg(g) +2SO_2 (g)_ + O_2_ | -111.41 | 216.3 |
| CaCO_3(s)_ = CaO(g) +CO_2(g)_ | -5.49 | 42.6 |


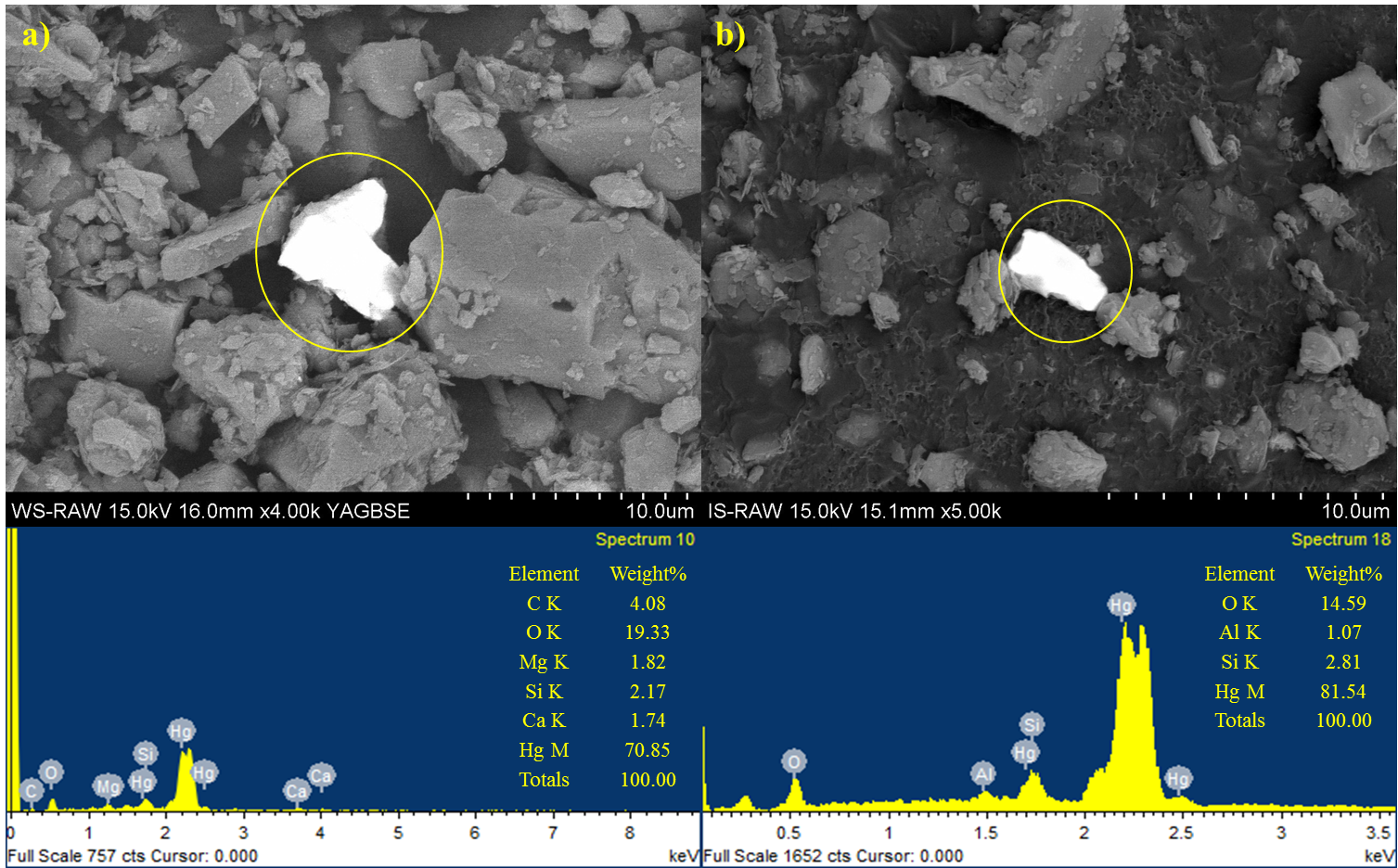


Fig. S1. SEM-EDS analysis of (a) mine soil and (b) industrial soil.


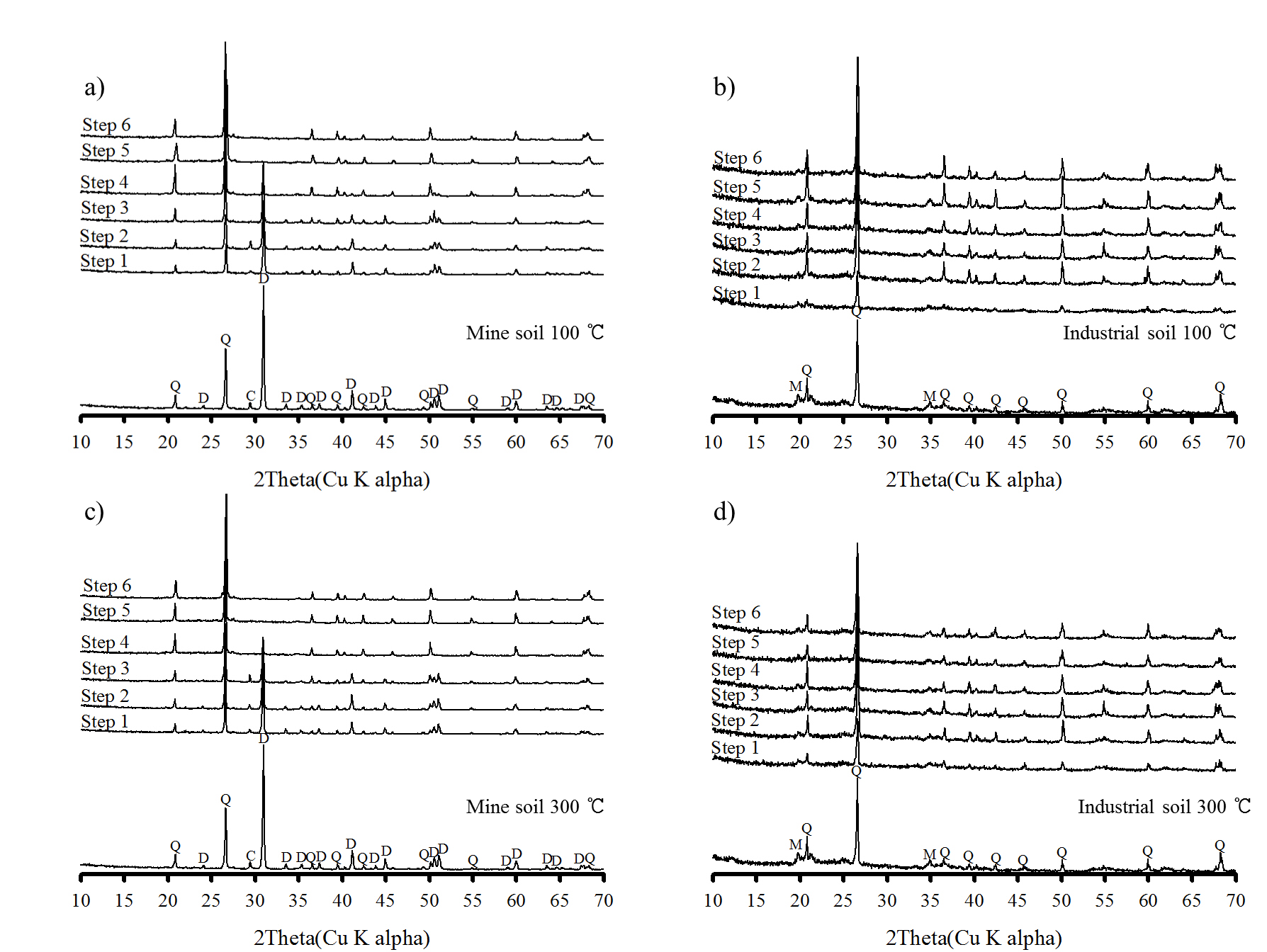


Fig. S2. X-ray diffraction patterns of Hg-contaminated soil samples and residual solid of soils after sequential extraction (after thermal treatment at 100 and 300 °C, for 30 min (a, c) mine soil and (b, d) industrial soil). (C: calcite; D: dolomite; M: muscovite; Q: quartz).


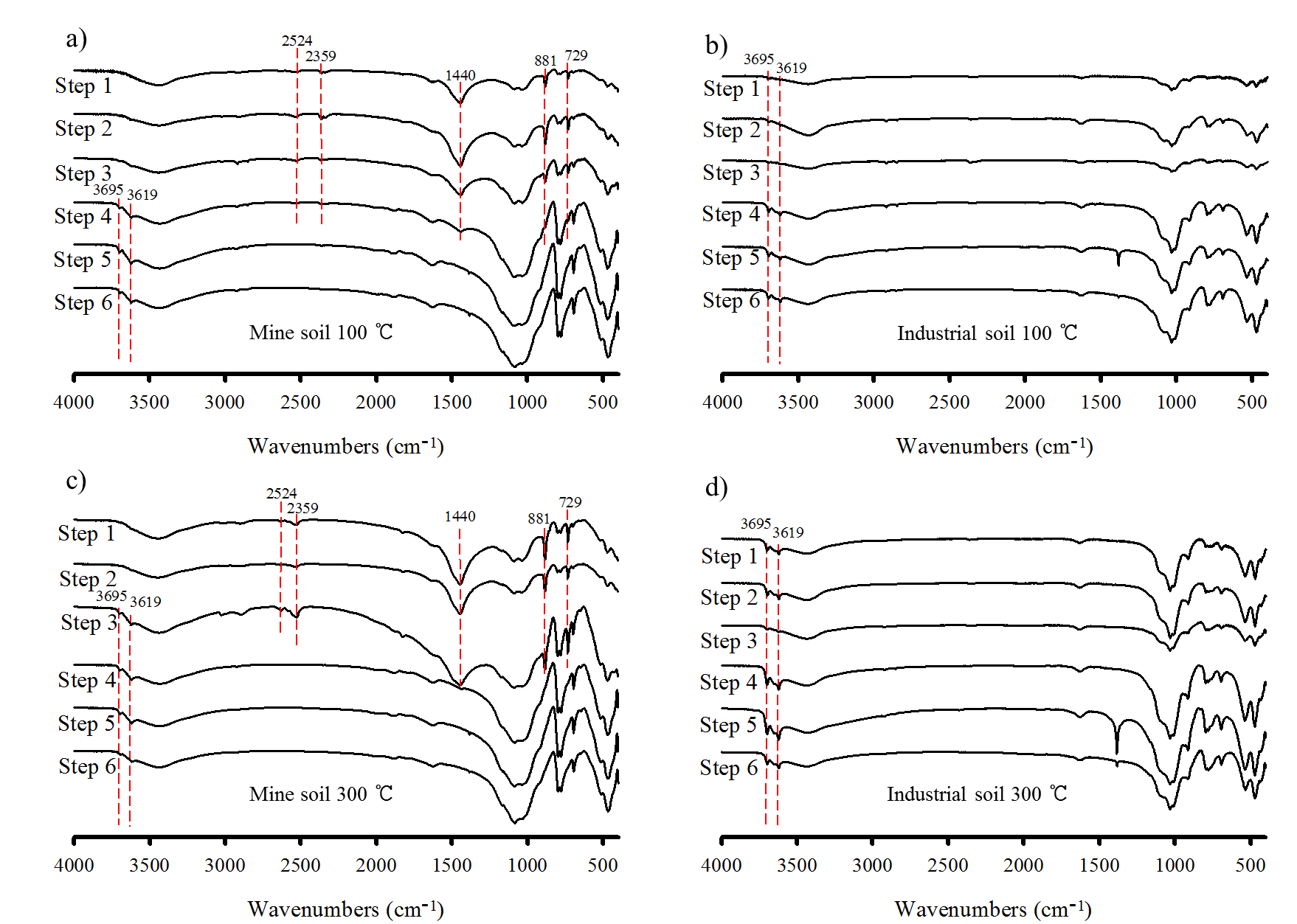


Fig. S3. FTIR spectra of residual solid of soils after sequential extraction (after thermal treatment at 100 and 300 °C, for 30 min (a, c) mine soil and (b, d) industrial soil).


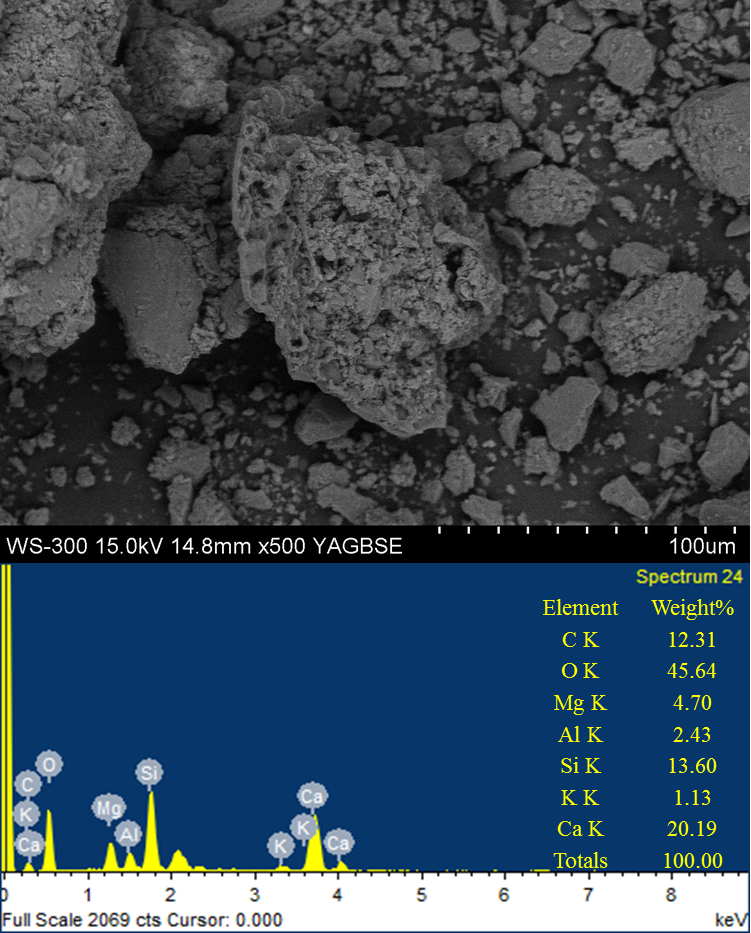


Fig. S4. SEM-EDS analysis of mine soil after thermal treatment at 300°C, for 30 min.


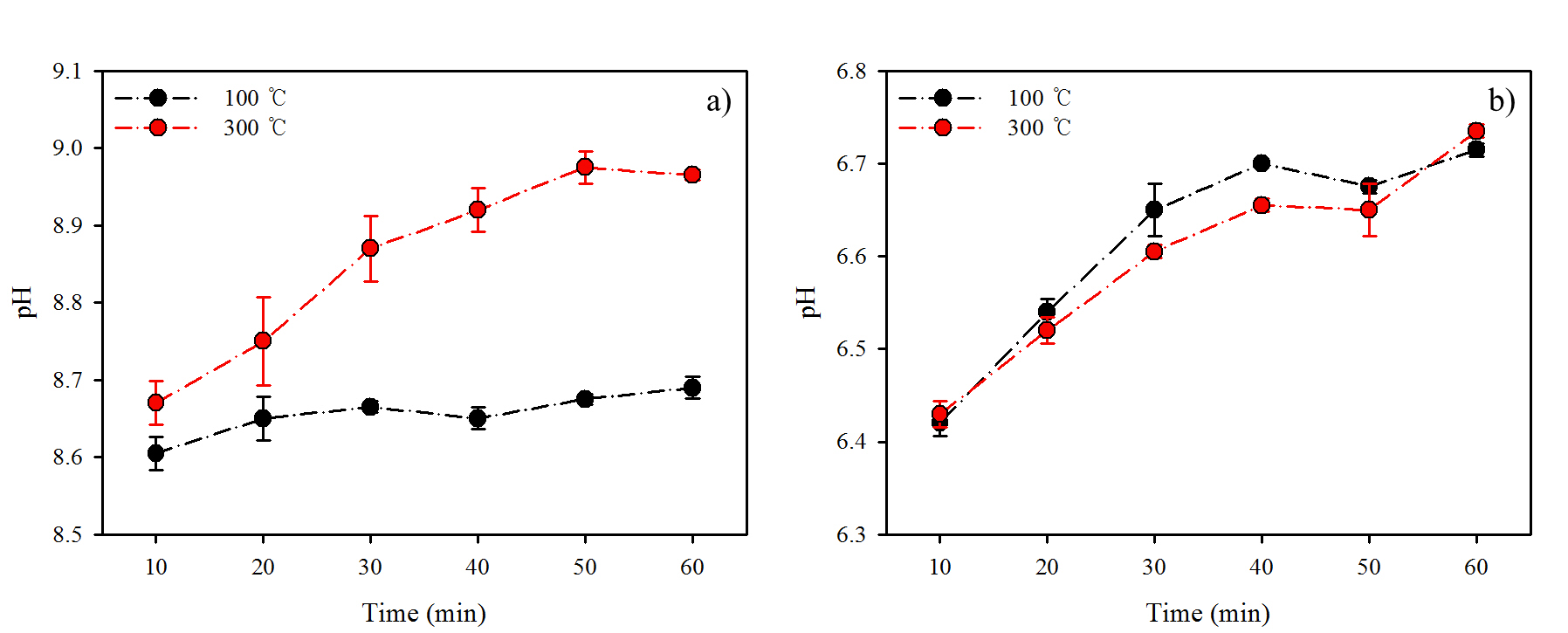


Fig. S5. Soil pH at 100 and 300 °C within 60 min of (a) mine soil and (b) industrial soil.
